# 2557 Improving ClinicalTrials.gov compliance: A coordinated effort for success

**DOI:** 10.1017/cts.2018.288

**Published:** 2018-11-21

**Authors:** Scott Patton, Elaine Basaca, Jennifer S. Brown

**Affiliations:** Stanford University School of Medicine

## Abstract

OBJECTIVES/SPECIFIC AIMS: ClinicalTrials.gov (CTgov) compliance has received much international attention as a significant regulatory, scientific, and ethical responsibility. Compliance rates for both industry and academia are held up for scrutiny by transparency advocates, but solutions for achieving compliance in academia have proven to be—because of its focus on innovation and multiple disciplines—significantly more complex than those employed by industry. Added challenges for academic medical centers (AMCs) are both increased researcher responsibilities under the new NIH Policy on Clinical Trial Dissemination and system-wide changes to requirements for “clinical trial only” Funding Opportunity Announcements. At Stanford University, a multifaceted approach toward improving CTgov outreach, education, and reporting led to a dramatic turnaround in compliance over 17-month period. METHODS/STUDY POPULATION: Stanford University School of Medicine’s Senior Associate Dean for Research and PI of Stanford’s CTSA applied a 3-part strategy to address unacceptable rates of results reporting. The strategy included (1) regular compliance reports to department chairs, (2) establishment of a central office, Clinical Research Quality (CRQ), to provide consistent training and support, and (3) interdepartmental cooperation across the school and university. Compliance reports, identifying all studies late for results reporting were sent monthly to all department chairs, with heightened focus on departments that conduct the most clinical trials. Senior leadership described the process in executive meetings and set improvement goals. Reports included multiple data points to help departments mobilize resources and identify trends; half-way through the period, soon-to-be late study records were included. CRQ hired 2 fulltime employees tasked with all aspects of managing the CTgov process and designed a portfolio of activities including: (1) a master list of all Stanford studies in the CTgov system; (2) a process for generating and distributing monthly reports; (3) an education program; and (4) support services, including an administrator working group. RESULTS/ANTICIPATED RESULTS: Since December 2015, Stanford has had the second-highest compliance rate improvement out of the 20 schools of medicine that receive the most NIH funding (+ 62%). DISCUSSION/SIGNIFICANCE OF IMPACT: Managing ClinicalTrials.gov compliance requires a high degree of technical knowledge of regulations, NIH policy, and the CTgov system. But without an equally high degree of engagement from senior leadership, results would not have been achieved. Central resources are critical to set policy and establish consistent processes, but without regular and repeated interactions between faculty, a multitude of administrators and staff, more central resources would have been required. By working simultaneously “down from the top” and “up from the bottom,” communication and education expanded rapidly, ineffective efforts were quickly transformed, and what began as an irritating and cumbersome problem became an occasion for collaboration and celebration of increased transparency.

